# Preclinical evaluation of radiolabeled tissue factor-targeted peptide for theranostics of hepatocellular carcinoma post percutaneous ethanol injection

**DOI:** 10.7150/thno.102130

**Published:** 2024-10-28

**Authors:** Buchuan Zhang, Pei Wang, Qiaorong Chen, Yilin Yang, Feng Xiong, Guangfa Bao, Jingfei Yang, Ziqiang Wang, Huimin Zhou, Shuang Song, Sijuan Zou, Dong-Hyun Kim, Bo Yu, Xiaohua Zhu

**Affiliations:** 1 Department of Nuclear Medicine, Tongji Hospital, Tongji Medical College, Huazhong University of Science and Technology, Wuhan, China.; 2 Department of Nuclear Medicine, Tongji Hospital, Tongji Medical College and State Key Laboratory for Diagnosis and Treatment of Severe Zoonotic Infectious Diseases, Huazhong University of Science and Technology, Wuhan, China.; 3 Department of Radiology, Feinberg School of Medicine, Northwestern University, Chicago, IL 60611, USA.

**Keywords:** HCC, Tissue factor, Percutaneous ethanol injection, PET imaging, Radiotherapy

## Abstract

**Rationale:** Tissue factor (TF) initiates local blood clotting and infiltration of tumor-associated macrophages, leading to tumor recurrence post-local ablation. Our study addressed inefficient cancer cell killing and immunosuppressive macrophage infiltration after percutaneous ethanol injection (PEI) in hepatocellular carcinoma (HCC). We evaluated the feasibility of ^18^F-radiolabeled polypeptide TF-targeted radioligand (tTF) as a PET tracer for assessing tumor response. We also explored the efficacy and safety of ^177^Lu-radiolabeled tTF to eradicate residual tumors and tumor-associated macrophages.

**Methods:** TF expression in the locally treated human HCC was assessed. Biodistribution, pharmacokinetics, and TF-targeted specificity of Al^18^F-NOTA-tTF were investigated in Kunming (KM) and/or Hepa1-6 mice. Evaluation of FDG/tTF PET imaging, histopathological characteristics, and tumor ablation response was conducted using two incomplete PEI ablation models, with ethanol volumes equivalent to 50% (high-dose (HD) PEI group) or 25% (low-dose (LD) PEI group) of the tumor volume administered. Following PEI, a single dose of ^177^Lu-DOTA-tTF was administered on day 1 to assess its efficacy in eradicating residual tumors and immunosuppressive macrophages. Systemic toxicity was evaluated through blood analysis and histological examination of healthy organs.

**Results:** Immunohistochemistry analysis demonstrated elevated TF expression around the ablation margin of residual tissue in human HCC. Radiolabeled tTF exhibited excellent TF-specificity, water solubility, and stability. FDG PET imaging and histological analysis showed tumor recurrence, upregulation of immunosuppressive macrophages, and TF around tumor foci post-treatment in the HD PEI-treated group. Meanwhile, the uptake of ^18^F-FDG exhibited a decline, while the uptake of Al^18^F-NOTA-tTF showed an increase in both the HD and LD PEI groups, as observed on day 1 and day 6 post-PEI. These results indicated that increased tTF uptake offers a specific and durable avenue for targeted theranostic applications. Following PEI, ^177^Lu-DOTA-tTF therapy demonstrated significant tumor suppression and eradication of immunosuppressive macrophages compared to control groups. Safety assessments indicated no significant toxicity in the main organs of tested animals.

**Conclusions:** Al^18^F-NOTA-tTF is a promising PET tracer for assessing ablated HCC, while ^177^Lu-DOTA-tTF provides an effective tool for inhibiting residual tumor growth and immunosuppressive macrophages post-PEI. Significantly, TF-targeting theranostics may help overcome incomplete cancer cell killing and formation of tumor immunosuppressive microenvironment, offering a promising strategy for effective HCC ablation in future clinical practice.

## Introduction

Hepatocellular carcinoma (HCC) is a prevalent malignancy globally and a leading cause of cancer-related mortality 1. Various ablation therapies, including ethanol, radiofrequency (RFA), Cryo, and irreversible electroporation (IRE) ablations, can achieve efficacy comparable to surgical resection for early-stage liver cancer and may render initially unresectable tumors amenable to surgery 2, 3. Especially, percutaneous ethanol injection (PEI) is recommended for early-stage HCC (≤2 cm) and lesions of 2-4 cm unsuitable for surgical resection due to anatomical or patient conditions 4, 5. Moreover, PEI is recognized in international guidelines as a curative treatment for very early and early-stage HCC 6. However, local ablations during aggressive tumor recurrence follow-up remain a concern, and the underlying mechanism post-PEI is not fully elucidated 7-9. Previous studies indicated that the residual tissue and tumor immunosuppressive microenvironment (TIME) contribute to tumor recurrence after ablation 10-12. Tumor-associated macrophages originating peripherally in TIME, often promoting pro-oncogenic signals for tumor recurrence and progression, are pivotal for immune tolerance and correlate with poor tumor prognosis 13, 14. Thus, finding markers associated with ablation therapies and modulating TIME is crucial for diagnosing and assessing post-ablation tumor responses and sequential combination therapies.

Tissue factor (TF) is a transmembrane protein that initiates the extrinsic coagulation pathway by binding and activating the proteinase factor VII/VIIa 15. It is expressed in various cell types, including cancer cells 15, 16, and plays a critical role in blood clotting 17. TF expression is ubiquitous in solid tumors and is associated with K-ras oncogene mutation, loss of p53 and PTEN tumor suppressor, and tumor hypoxia 18, 19. In liver cancer, TF contributes to angiogenesis and invasiveness, and its expression serves as a potential prognostic indicator. Low levels of TF expression correlate with enhanced overall survival in HCC patients 20, 21. Furthermore, TF expression is predominantly localized to damaged tissues, initiating the extrinsic coagulation pathway in response to external damage. Therefore, TF-targeting strategies are highly specific for monitoring and treating ablated regions 22. Given these findings, TF is a promising target for effectively diagnosing and treating HCC.

To overcome incomplete cancer cell killing and the presence of immunosuppressive macrophages, we propose a theranostics strategy utilizing a polypeptide targeting tissue factor (tTF) for PET imaging and localized internal radiotherapy following PEI treatment. We evaluated the feasibility of ^18^F-radiolabeled tTF as a PET tracer for assessing HCC response post-PEI and explored the efficacy and safety of ^177^Lu-radiolabeled tTF radiotherapy in targeting both residual tumors and tumor-associated macrophages post-PEI.

## Materials and Methods

The Supplementary Materials provide comprehensive details regarding all chemicals, reagents, and solvents. The section also includes additional information on cell culture methods and establishing the tumor-bearing mouse model.

### Pathological analysis of human liver cancer

The liver cancer samples were collected from patients at Tongji Hospital, Tongji Medical College, Huazhong University of Science and Technology. This study was approved by the Clinical Research Ethics Committee of Tongji Hospital, which is affiliated with Tongji Medical College, Huazhong University of Science and Technology. Immunohistochemistry (IHC) was performed according to the authorized clinical protocol. Ten patients received TACE or microwave coagulation interventional therapy. Pathological analysis supported the diagnosis of liver cancer in all cases. IHC was performed using a standard protocol for the TF. The proportion of staining-positive area was analyzed by semi-quantitative analysis.

### Radiolabeling and quality control

The radiolabeling of Al^18^F-NOTA-tTF, ^68^Ga-DOTA-tTF, and ^177^Lu-DOTA-tTF was performed according to previously reported methods 23-25. Details of the radiolabeling process can be found in the Supplemental Materials.

### *In vivo* evaluation of Al^18^F-NOTA-tTF

We investigated the biodistribution and pharmacokinetics of Al^18^F-NOTA-tTF in Kunming mice (KM); detailed information is available in Supplemental Materials.

### PEI treatment and efficacy monitoring

In this study, we utilized Hepa1-6 tumor-bearing mice to establish two ablation models of incomplete ablation of liver cancer via PEI. The high-dose ethanol injection (half of the tumor volume) was the treatment group (HD-PEI group), and the low-dose ethanol injection (a quarter of the tumor volume) was the treatment group (LD-PEI group). Detailed procedural steps are provided in Supplementary Materials.

### Al^18^F-NOTA-tTF PET imaging and biodistribution in the HD-PEI model

We randomly divided tumor-bearing mice into the HD-PEI treatment (HD-PEI, n = 8), sham treatment (Sham, n = 8), and negative control groups (NC, n = 6). Al^18^F-NOTA-tTF PET/CT imaging was conducted on days 1 and 6 post-PEI. Hepa1-6 tumor-bearing mice received an intravenous injection of 100 μL of Al^18^F-NOTA-tTF (2.96 MBq). Tumor volume and mouse body weight were monitored every 2 to 3 days. Biodistribution studies were performed on days 1 and 6 post-PEI, with mice divided into PEI and NC groups. Al^18^F-NOTA-tTF (1.2 MBq) was administered via tail vein injection, and biodistribution results were expressed as the percentage of injected dose per gram (%ID/g).

### PET imaging in the HD-PEI and LD-PEI models

Tumor-bearing mice were randomly divided into the HD-PEI treatment (HD-PEI, n = 8), LD-PEI treatment (LD-PEI, n = 8), and negative control (NC, n = 8) groups. Al^18^F-NOTA-tTF PET/CT imaging was conducted on days 1, 5, and 12 post-PEI.^ 18^F-FDG PET/CT imaging was conducted on days 2, 6, and 13 post-PEI. Hepa1-6 tumor-bearing mice received intravenous injections of 100 μL of Al^18^F-NOTA-tTF (2.96 MBq) and^ 18^F-FDG (2.96 MBq). Tumor volume and mouse body weight were monitored every 2 days.

### Radioligand therapy studies

PEI therapy was initiated when the tumor volume reached 100 mm^3^. Mice were randomly assigned to five groups: PEI (group 1, n = 10), PEI + 0.45 mCi ^177^Lu-DOTA-tTF (group 2, n = 10), PEI + 0.75 mCi ^177^Lu-DOTA-tTF (group 3, n = 8), 0.45 mCi ^177^Lu-DOTA-tTF (group 4, n = 10), and saline (group 5, n = 10). A single dose of^ 177^Lu-DOTA-tTF was administered on day 1 post-PEI. Tumor volume, body weight, and survival were monitored every 3 days. ^18^F-FDG PET/CT imaging was performed on days 3, 7, 13, and 22 after PEI to assess therapeutic efficacy. Mice were euthanized upon reaching a tumor volume of 1000 mm^3^ or upon observation of tumor ulceration. Safety and tolerability assessments were conducted at the endpoint of radiation therapy.

### Histological staining

For the imaging study, tumor tissues were collected on days 3 and 6 after PEI, and histological analysis was performed. For the radiotherapy study, tumor tissues collected on day 4 after administration of ^177^Lu-DOTA-tTF and at the endpoint were also subjected to histological analysis. Histological assessments included hematoxylin-eosin (H&E) staining of the liver, spleen, and kidney. Immunohistochemistry (IHC) was performed to evaluate the expression of Ki-67, TF, F4/80, CD86, CD163, and H2AX in the tumor tissues.

### Statistical analysis

All quantitative data were presented as mean ± standard deviation (SD). Statistical analysis was performed using the Student's *t*-test and one-way ANOVA by GraphPad Prism version 9.0. *P* values less than 0.05 were considered statistically significant.

## Results

### Pathological analysis of the residual human liver cancer

**Figure 1:**
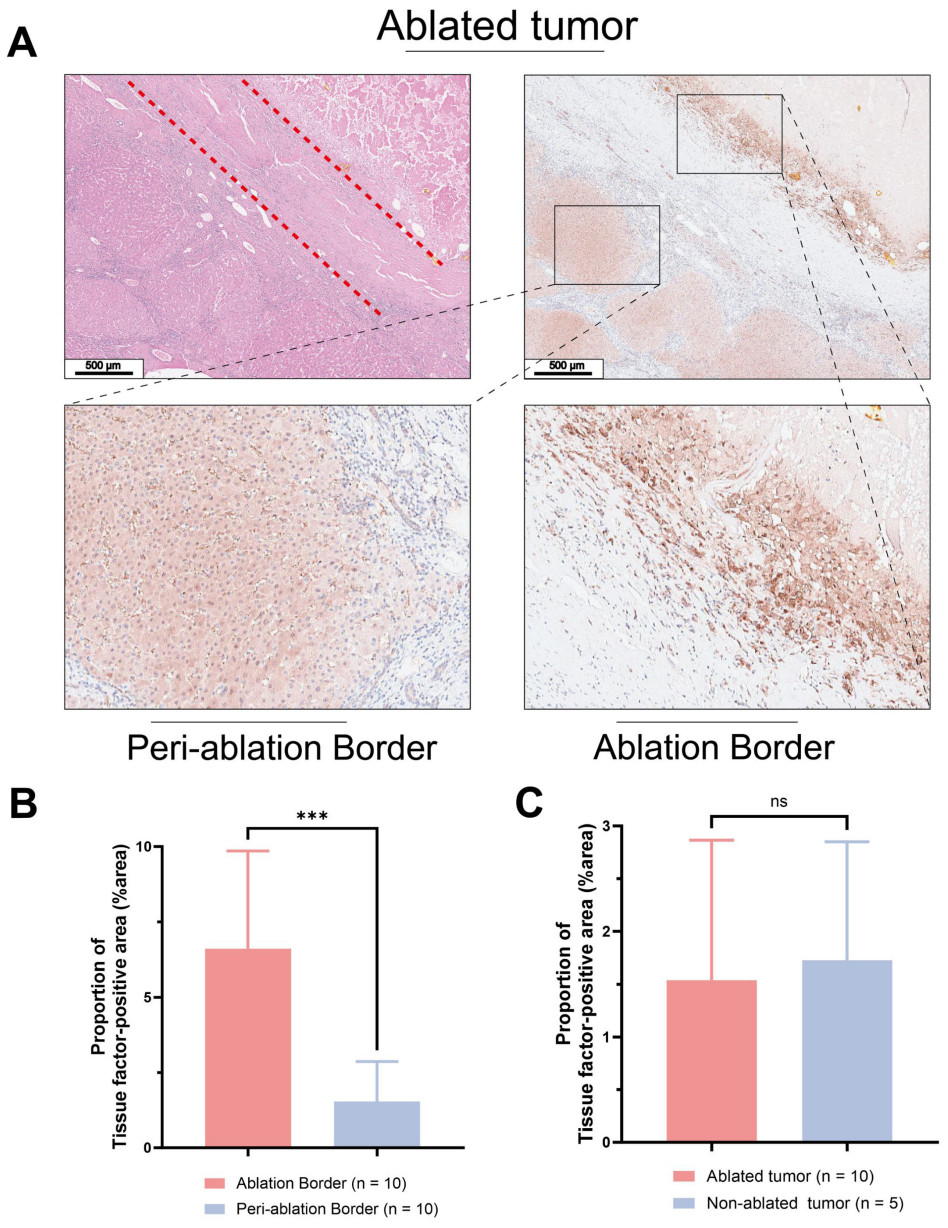
** Pathological analysis of treated human liver cancer.** H&E and IHC using anti-TF antibody of **(A)** Human liver cancer specimens (Scale bar = 500 μm). **(B)** Patients who underwent ablation. The TF at the border of the ablation area was significantly higher than that in the tumor tissue distal to the ablation area. (6.61 ± 3.25 vs. 1.54 ± 1.33, P < 0.05).** (C)** No significant difference in TF expression between the distal tumor ablation area and the tumor tissue of patients without ablation (1.54 ± 1.33 vs. 1.73 ± 1.13, P > 0.05).

**Figure 1A** displays histological sections of human liver cancer post-local ablative interventional therapy, stained with H&E and anti-TF. H&E staining delineated a distinct border between the ablated area and residual tumor tissue. **Figures 1B & C** show intense brown IHC staining, indicating high TF expression at the ablation border compared to the periphery of the ablation zone ((%area): 6.61 ± 3.25 vs. 1.54 ± 1.33, P < 0.05). There was no statistically significant difference in TF expression levels between the periphery of the ablation zone and the tumor tissue of patients who did not undergo ablation treatment ((%area): 1.54 ± 1.33 vs. 1.73 ± 1.13, P > 0.05).

### Radiolabeling and quality control

**Figure 2:**
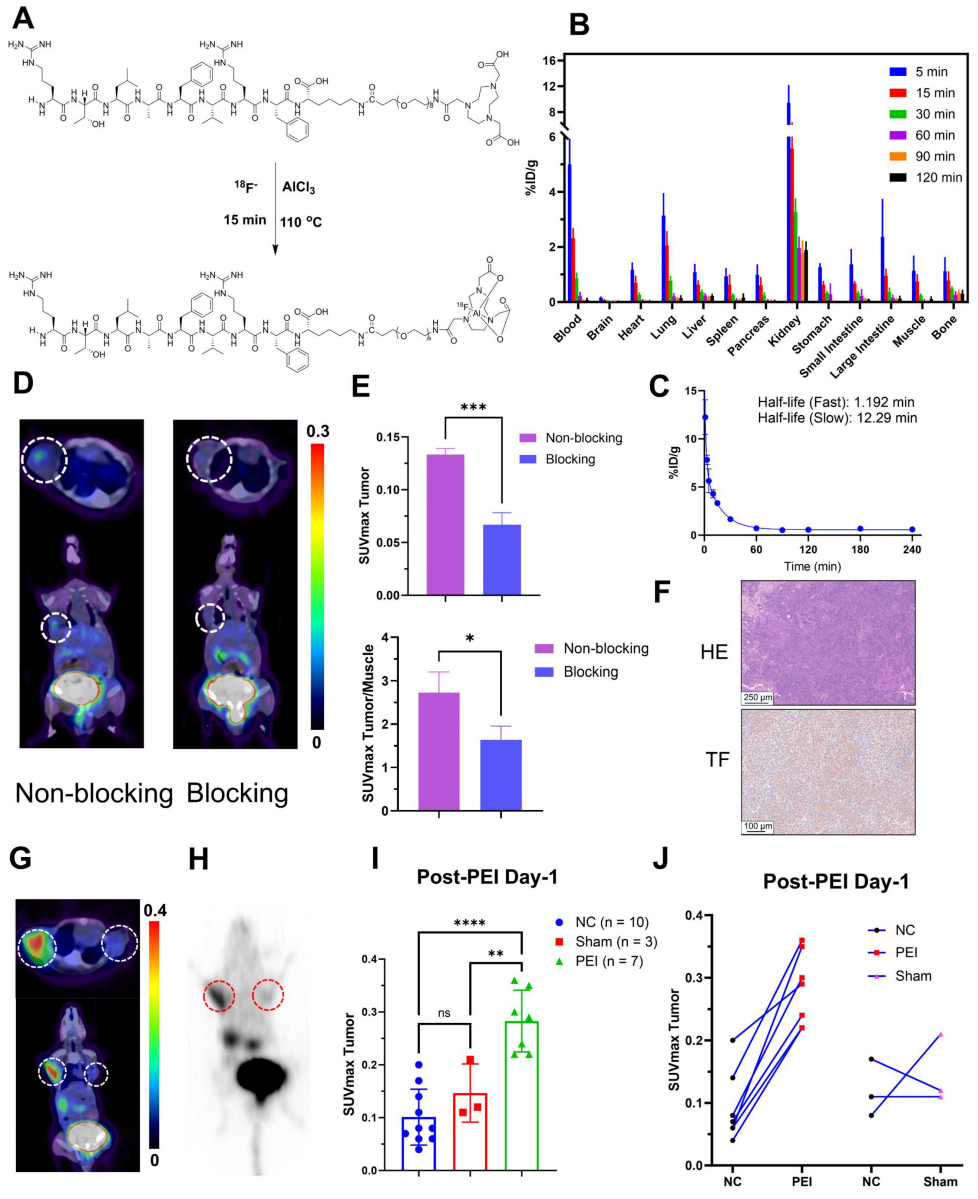
** Preparation and biological evaluation of radiolabeled probes. (A)** Schematic illustration of the synthesis of the Al^18^F-NOTA-tTF. **(B)** Biodistribution of Al^18^F-NOTA-tTF in KM mice at different time points postinjection (n = 5). **(C)** Pharmacokinetic study of Al^18^F-NOTA-tTF in KM mice (n = 5).** (D & E)** Representative PET imaging and quantification results of Al^18^F-NOTA-tTF in Hepa1-6 and blocked Hepa1-6 tumor-bearing mice. **(F)** IHC of Hepa1-6 xenografts with TF expression. **(G & H)** Representative PET imaging and maximum intensity projection (MIP) of ^68^Ga-DOTA-tTF in Hepa1-6 tumor-bearing mice (left circle: PEI; right circle: NC). **(I & J)** Quantification of PET imaging of ^68^Ga-DOTA-tTF in Hepa1-6 tumor-bearing mice.

**Figure 2A** depicts the structures and labeling process of Al^18^F-NOTA-tTF. The synthesis was completed manually within 40 minutes, achieving a non-decay-corrected radiochemical yield of 22.5 ± 4.7% (n = 10). Specific activity ranged from 8.8 to 23.4 MBq/nmol (n > 10). The quality control results are detailed in **Table 1** and **Figure S1**.

The radiochemical purity of Al^18^F-NOTA-tTF, ^68^Ga-DOTA-tTF, and ^177^Lu-DOTA-tTF was over 99%, as determined by radio-TLC (**Figure S1A, B, H**). Al^18^F-NOTA-tTF and^ 68^Ga-DOTA-tTF remained stable during *in vitro* testing in saline or fetal bovine serum, showing a single peak after incubation in the TLC chromatogram assay (**Figure S1C-F**). The log P values of Al^18^F-NOTA-tTF and ^68^Ga-DOTA-tTF were -3.07 ± 0.10 and -2.72 ± 0.04, indicating high hydrophilicity (**Figure S1G**).

### *In vivo* pharmacokinetic evaluation of Al^18^F-NOTA-tTF

In KM mice, Al^18^F-NOTA-tTF demonstrated minimal uptake in normal organs, such as the liver, brain, spleen, and pancreas, and showed rapid renal clearance with a sharp decline over time (**Figure 2B**). Pharmacokinetic analysis confirmed the rapid elimination of the tracer characterized by a short distribution phase half-life of 1.192 min and an elimination phase half-life of 12.29 min in KM mice (**Figure 2C**).

The PET/CT imaging of Al^18^F-NOTA-tTF is shown in Figure 2D, demonstrating tumor uptake in Hepa1-6 tumor-bearing mice at 60 min post-injection, along with high radioactive signals in the bladder. In the blocking study (**Figure 2E**), pretreatment with 0.2 mg of the unlabeled precursor successfully reduced Hepa1-6 tumor uptake compared to the non-blocked condition (SUVmax: 0.07 ± 0.010 vs. 0.13 ± 0.005, P < 0.05, n = 3). Dynamic PET/CT imaging and biodistribution studies confirmed the effective tumor detection capability of Al^18^F-NOTA-tTF. Histological analysis further validated TF expression in Hepa1-6 tumors (**Figure 2F**).

We also established a bilateral Hepa1-6 tumor-bearing mouse model to evaluate targeted TF-specific imaging post-PEI. PET imaging showed that 60 min after injection, the SUVmax of tumors in the PEI group (n = 7) was significantly higher than that in the Sham (n = 3) and NC (n = 10) groups (0.28 ± 0.06 vs. 0.15 ± 0.06 vs. 0.10 ± 0.05, P < 0.05) (**Figure 2G-I**). A significant upward trend was observed in the tumors on the PEI-treated side compared with the control side, but no such trend existed in the sham-treated group (**Figure 2J**, **Figure S3C**). These results illustrated the feasibility of ^68^Ga-DOTA-tTF in evaluating the ablation response of hepatocellular carcinoma.

### PEI treatment and ablation response monitoring

**Figure 3:**
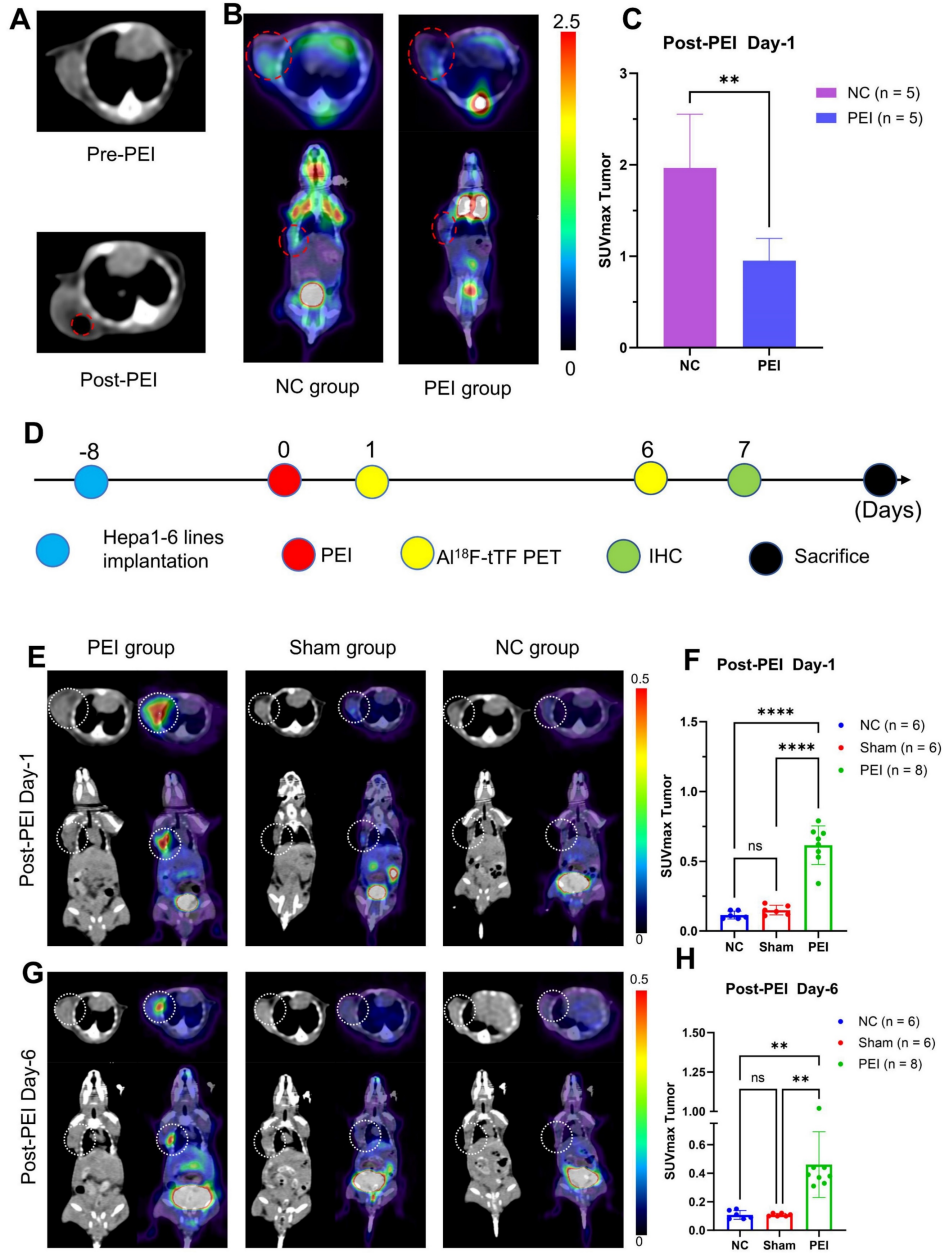
** Monitoring PEI efficacy by Al^18^F-NOTA-tTF PET imaging post-PEI. (A)** Representative CT scans pre- and post-PEI. **(B & C)** Representative ^18^F-FDG PET/CT imaging and quantification results post-PEI. **(D)** PEI treatment and PET imaging schedule in Hepa1-6 tumor-bearing mice. **(E & F)** Representative PET imaging and quantification results of Al^18^F-NOTA-tTF in Hepa1-6 subcutaneous xenograft models on day 1 post-PEI. **(G & H)** Representative PET imaging and quantification results of Al^18^F-NOTA-tTF in Hepa1-6 subcutaneous xenograft models on day 6 post-PEI. White circles indicate tumors.

CT scanning and ^18^F-FDG PET/CT imaging were employed on day 1 post-PEI to assess PEI ablation response (**Figure 3A-C**). CT images revealed a low-density shadow indicative of ethanol distribution, distinguished by its lower CT value than the tumor tissue (**Figure 3A**). ^18^F-FDG PET/CT imaging **(Figure 3B-C)** demonstrated significantly reduced tumor uptake in the PEI group compared to the controls (SUVmax: 0.95 ± 0.24 vs. 1.96 ± 0.59, P = 0.0074 < 0.05), indicating the effectiveness of PEI treatment.

Biochemical indexes of routine blood analysis on days 2 and 10 post-PEI showed no notable abnormalities (**Figure S2A**), and histological examination of vital organs exhibited no signs of hepatotoxicity or tubular injury (**Figure S2B**).

PET imaging was conducted on days 1 and 6 post-PEI to evaluate the feasibility of Al^18^F-NOTA-tTF for assessing the PEI ablation response (**Figure 3D-H**). On day 1, Al^18^F-NOTA-tTF PET imaging revealed that the SUVmax of tumor uptake in the PEI group (n = 8) was 0.62 ± 0.14 at 60 min post-injection, significantly higher than that in the Sham and NC groups (0.15 ± 0.04, 0.12 ± 0.03, n = 6) (**Figure 3E-F**). Similarly, biodistribution analysis on day 1 indicated significantly elevated tumor uptake in the PEI group compared to the NC group at 60 min post-injection (0.77 ± 0.25 vs. 0.40 ± 0.03 %ID/g, P < 0.05, n = 4) (**Figure S3A**).

On day 6, PET imaging revealed a significantly higher SUVmax of tumor uptake in the PEI group (n = 8) compared to the Sham (n = 6) and NC groups (n = 6) (0.46 ± 0.23 vs. 0.11 ± 0.01 vs. 0.11 ± 0.03, P < 0.05) (**Figure 3 G-H**). Biodistribution analysis on day 6 showed that tumor uptake in the PEI group (n = 3) was markedly higher than in the NC group (n = 4) at 60 minutes post-injection (0.50 ± 0.28 vs. 0.13 ± 0.01 %ID/g, P < 0.05) (**Figure S3B**). Additionally, the tumor volume in the PEI group on day 7 was significantly smaller than in the NC group (**Figure S4**), indicating effective tumor suppression after PEI treatment.

**Figure 4:**
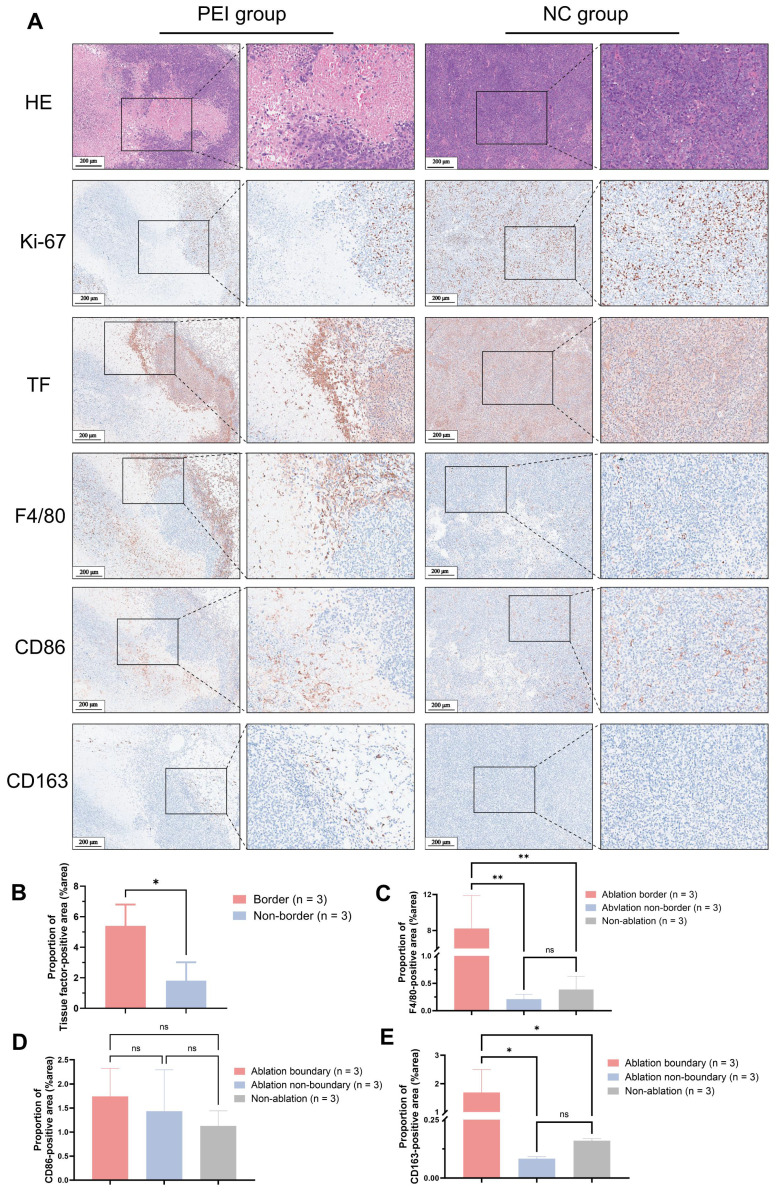
** Histopathological results of tumor from Hepa1-6 tumor-bearing mice on day 3 post-PEI. (A)** H&E and IHC of Ki-67, TF, F4/80, CD86, and CD163. **(B-F)** 400× visual fields were imaged randomly to quantify TF, F4/80, CD86, and CD163. (Scale bar = 200 μm).

Histopathological analysis of mice on day 3 post-PEI revealed extensive tumor cell necrosis within the ablation area and a distinct border between the ablated region and residual tumor tissue (**Figure 4A**). High expression of TF was explicitly observed at the ablation border (proportion of TF-positive area (%area): 5.40 ± 1.39 vs. 1.81 ± 1.21, P < 0.05) (**Figure 4B**). Elevated levels of F4/80 and CD163 at the ablation border (F4/80(%area): 8.22 ± 3.67 vs. 0.21 ± 0.09 vs. 0.39 ± 0.24, P < 0.05; CD163(%area): 1.69 ± 0.81 vs. 0.08 ± 0.01 vs. 0.16 ± 0.01, P < 0.05) were observed by immunohistochemical staining, whereas CD86 expression showed no significant difference between the border and non-border areas of residual tumors ((%area): 1.74 ± 0.58 vs. 1.43 ± 0.86 vs. 1.13 ± 0.31, P > 0.05) (**Figure 4C-E**). Similar findings were observed in mice on day 6 after PEI surgery (**Figure S5**).

### PET imaging in HD-PEI and LD-PEI models

**Figure 5:**
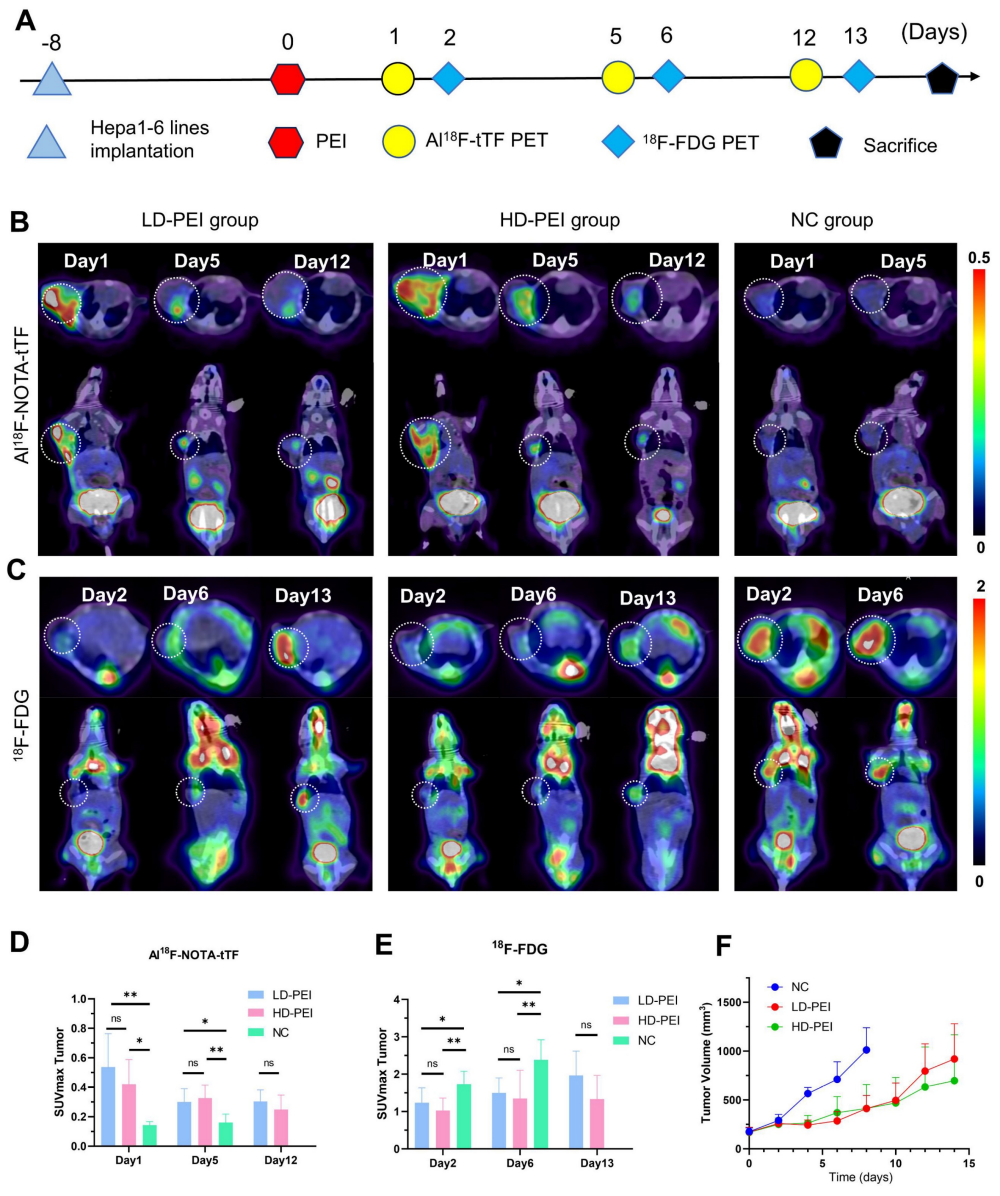
** Evaluation of Al^18^F-NOTA-tTF in two incomplete ablation models. (A)** PEI treatment and PET imaging schedule in Hepa1-6 tumor-bearing mice. **(B)** Representative PET imaging of Al^18^F-NOTA-tTF on days 1, 5, and 12 of post-PEI and **(C)** ^18^F-FDG on days 2, 6, and 13 post-PEI in Hepa1-6 tumor-bearing mice. **(D)** Quantification results of Al^18^F-NOTA-tTF and **(E)** ^18^F-FDG post-PEI in Hepa1-6 tumor-bearing mice. **(F)** Tumor growth curves. White circles indicate tumors.

To explore the sensitivity of Al^18^F-NOTA-tTF in assessing ablation response after PEI, we injected in two ablation models 50% (HD PEI group) or 25% (LD PEI group) volume of tumor size of ethanol and performed PET imaging over an extended observation period (**Figure 5A**). Al^18^F-NOTA-tTF PET/CT imaging was conducted on day 1, day 5, and day 12 post-PEI (**Figure 5B**). On days 1 and 5, PET imaging showed that the SUVmax of tumor uptake in the HD-PEI group and LD-PEI group was significantly higher than the NC group (day 1: 0.42 ± 0.17 vs. 0.54 ± 0.23 vs. 0.12 ± 0.04, P < 0.05; day 5: 0.33 ± 0.09 vs. 0.30 ± 0.09 vs. 0.16 ± 0.06, P < 0.05) (**Figure 5D**). No significant difference was observed in tumor SUVmax uptake between the HD-PEI and LD-PEI groups on days 1 and 5. On day 12, tumor SUVmax did not differ significantly between the HD-PEI and LD-PEI groups (0.25 ± 0.10 vs. 0.31 ± 0.08, P = 0.2232 > 0.05) (**Figure 5D**). These PET results highlighted the sensitivity of Al^18^F-NOTA-tTF in assessing ablation response in different ablation models. Tumor uptake was comparable between the HD-PEI and LD-PEI groups, highlighting the potential of Al^18^F-NOTA-tTF in assessing ablation response in small-size HCCs. Al^18^F-NOTA-tTF PET imaging also showed that the tumor uptake decreased over time after PEI treatment, indicating that this probe had a window period for monitoring ablation response.

^18^F-FDG PET/CT imaging was conducted on days 2, 6, and 13 post-PEI (**Figure 5C**). PET imaging on days 2 and 6 showed that the tumor uptake SUVmax in the NC group was higher than that in the HD-PEI and LD-PEI groups (day 2: 1.74 ± 0.34 vs. 1.02 ± 0.34 vs. 1.23 ± 0.40, P < 0.05; day 6: 2.38 ± 0.54 vs. 1.35 ± 0.75 vs. 1.50 ± 0.40, P < 0.05) (**Figure 5E**). On day 13, there was no significant difference in tumor SUVmax between the HD-PEI and LD-PEI groups (1.33 ± 0.64 vs. 1.96 ± 0.65, P = 0.0957 > 0.05). Interestingly, ^18^F-FDG PET/CT imaging revealed an opposite trend to Al^18^F-NOTA-tTF in PEI-treated tumors of an increased tumor uptake over time post-PEI, suggesting recurrence of the residual tumor as demonstrated by tumor growth curves shown in **Figure 5F**.

### Radioligand therapy

**Figure 6:**
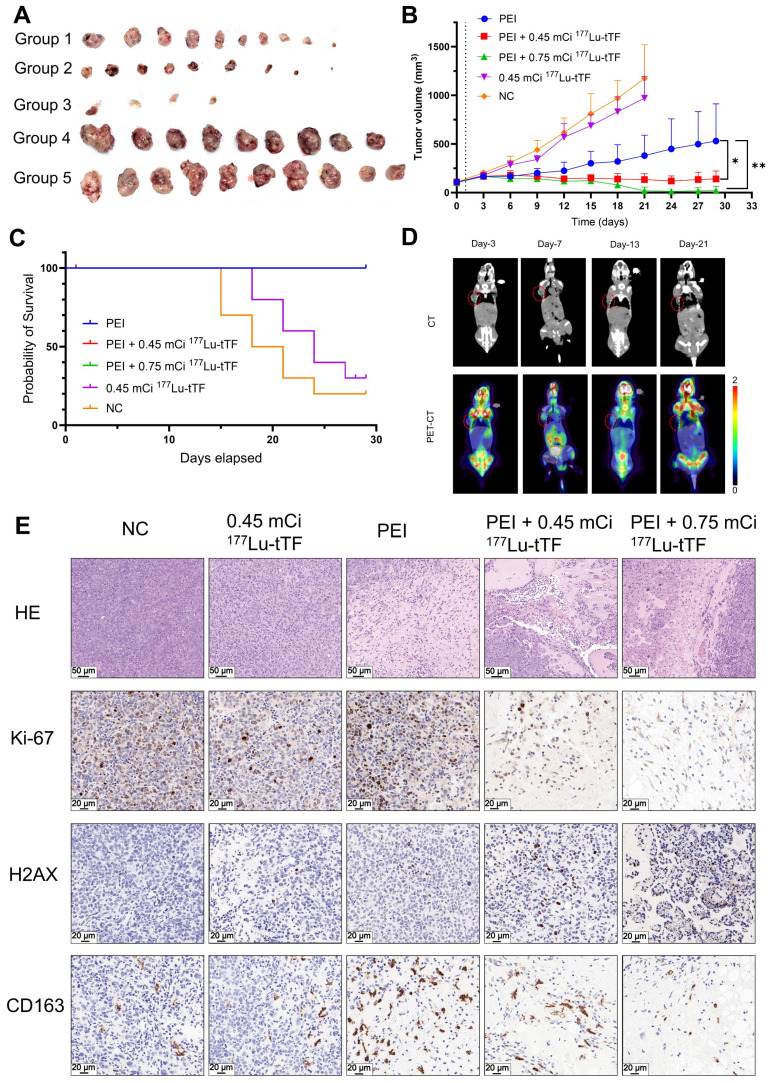
** Radioligand therapy efficacy of single-dose ^177^Lu-DOTA-tTF post-PEI in Hepa1-6 tumor-bearing mice. (A)** Photos of the tumor tissue of each group at the end of the therapy. **(B)** Tumor volume curves of each group within 29 days after the therapy started. **(C)** Kaplan-Meier survival curves of each group. **(D)** Representative ^18^F-FDG PET/CT imaging of the same tumor-bearing mouse on day 3, day 7, day 13, and day 21 after PEI in the PEI combined with 0.75 mCi ^177^Lu-DOTA-tTF group and SUVmax of tumor areas 0.68, 1.51, 0.57, 0.52, respectively. **(E)** H&E (scale bar = 50 μm) and IHC of Ki-67, H2AX and CD163 (scale bar = 20 μm) on day 3 after^ 177^Lu-DOTA-tTF administration.

In radioligand therapy, tumor growth was markedly inhibited in the PEI + 0.45 mCi and PEI + 0.75 mCi ^177^Lu-DOTA-tTF groups compared to the PEI group (**Figure 6A-B**). Median survival rates were 19.5 days and 24 days in the control and 0.45 mCi ^177^Lu-DOTA-tTF groups, respectively (**Figure 6C**). Notably, complete tumor disappearance occurred in three mice from the PEI + 0.75 mCi ^177^Lu-DOTA-tTF group. ^18^F-FDG PET/CT imaging of one of these mice confirmed tumor disappearance (SUVmax: day 3: 0.68, day 7: 1.51, day 13: 0.57, day 21: 0.52) (**Figure 6D**).

In the radioligand therapy study, Ki-67 and CD163 expression decreased, but H2AX expression increased in the PEI combined with the radiation therapy group starting from day 3 post-administration (**Figure 6E**). These data suggested that PEI combined with radiation therapy inhibits tumor proliferation and reduces M2-type macrophage infiltration.

### Evaluation of safety and tolerability of radiation therapy

There were no significant differences in the health status and body weight of mice among the treatment groups during the experiment. Blood tests after the study indicated no signs of blood, renal, or liver toxicity across all groups. Furthermore, the evaluation of safety and tolerability did not reveal any toxicity affecting healthy organs (**Figure S6-7**).

## Discussion

Recent studies have shed light on how tumor immune microenvironment (TIME) changes following incomplete ablation contribute to tumor progression 11, 12, 26. In mouse models of hepatocellular carcinoma and colorectal tumors, incomplete radiofrequency ablation (iRFA) has been shown to promote M2-like macrophage infiltration while suppressing T-cell function 11, 26. One study reported the development of a hydrogel based on STING (stimulator of interferon genes) agonists to promote immune activation, thereby enhancing the efficacy of radiofrequency ablation for hepatocellular carcinoma and effectively inhibiting the proliferation and metastasis of residual tumors caused by incomplete radiofrequency ablation 26. Despite various efforts to eradicate residual tumors and prevent recurrence, the progress made so far remains limited. Moreover, few studies have reported changes in TIME following PEI in HCC.

PEI is an essential clinical practice in hepatocellular carcinoma treatment, particularly for high-risk sites near vital organs (the gallbladder, stomach, colon, or nearby other organs) 6, 27. However, long-term follow-up has revealed invasive tumor recurrence post-PEI 8, 28. Although combining PEI with different modalities, such as chemotherapy, radiotherapy, and immunotherapy, can improve long-term HCC prognosis 29-34, the complexity of incomplete ablation and TIME leads to post-PEI recurrence. Therefore, monitoring tumor response and treating residual sites post-PEI are critical for effective tumor management.

### Local treatment triggered upregulation of the targeting pool with high specificity

IHC analysis of human liver cancer post-ablation revealed a high expression of TF at the ablation zone boundary (**Figure 1A**). This finding identified an ablation-triggered target pool in residual tumors and TIME following incomplete ablation of hepatocellular carcinoma. Furthermore, given the infiltration of immune cells from peripheral blood towards the ablation zone, the colocation of TF and immune cells could contribute to TIME modulation.

A tumor recurrence model was established by incomplete PEI ablation of HCC (**Figure 3A-C** and **Figure 5**) to explore the pathological features (**Figure 4**). As shown in **Figure 4A**, histopathological analysis of mice on day 3 post-PEI revealed significantly high TF expression and a distinct border between the ablated region and residual tumor. IHC staining of F4/80 and CD163 indicated elevated levels of tumor-associated macrophages at the ablation border. Also, CD86 expression showed no significant difference between border and non-border areas of residual tumors. Similar findings were observed in mice on day 6 after PEI surgery (**Figure S5**). Thus, PEI ablation triggered the upregulation of TF, identifying a specific site for follow-up imaging and treatment. Significantly, the colocation of TF and macrophages provides an opportunity for simultaneously eradicating residual tumor and immunosuppressive cells.

### TF-targeted PET imaging monitored tumor ablation response

A radiolabeled polypeptide targeting TF (tTF) as a PET tracer was proposed to eradicate residual tumors and immunosuppressive macrophages simultaneously and monitor tumor response post-PEI. Radiolabeled tTF exhibited water solubility and stability (**Figure 2** and **Table 1**). The evaluation of *in vivo* pharmacokinetics of Al^18^F-NOTA-tTF in KM mice revealed a biphasic decay and excellent excretion from normal organs. In Hepa1-6 tumor-bearing mice, Al^18^F-NOTA-tTF PET imaging showed tumor accumulation compared to other organs, with rapid clearance from the body. Blocking studies, dynamic PET/CT imaging and biodistribution studies further confirmed the detection capabilities of Al^18^F-NOTA-tTF.

To explore the feasibility of Al^18^F-NOTA-tTF for assessing the ablation response following PEI, PET/CT imaging (**Figure 3E-H**) and *ex vivo* biodistribution (**Figure S3**) were conducted on days 1 and 6 post-PEI, revealing persistent elevation of TF up to day 6 post-PEI, corroborated by immunohistochemistry (**Figure S5**). Moreover, on day 7 post-PEI, tumor volume was significantly smaller in the PEI group than in the NC group (**Figure S4**), underscoring the efficacy of PEI treatment while highlighting the potential for tumor recurrence based on observed volume changes.

By comparing with ^18^F-FDG PET, we further investigated the sensitivity and potential application of Al^18^F-NOTA-tTF in assessing the ablation response post-PEI. Using liver cancer models treated with high-dose (HD) and low-dose (LD) ethanol injections, we conducted longitudinal imaging studies (**Figure 5**). The decreased uptake of ^18^F-FDG and increased uptake of Al^18^F-NOTA-tTF lasted for 6 days post-PEI in both HD and LD PEI groups, indicating that Al^18^F-NOTA-tTF can effectively evaluate the ablation response in varying degrees of ethanol ablation. Furthermore, the consistently increased uptake of tTF endowed a highly specific and sustained route for follow-up theranostics.

### TF-targeted internal radiotherapy simultaneously eradicated residual tumor and immunosuppressive cells

TF has been shown to initiate local coagulation, contributing to tumor recurrence through the intra-tumoral recruitment of tumor-associated macrophages and extracellular matrix remodeling 35. IHC analysis of mouse tumors on days 3 and 6 post-PEI demonstrated significantly elevated levels of F4/80 and CD163 at the ablation area boundary compared to the control group, suggesting increased presence of tumor-associated macrophages, particularly M2 macrophages, which co-localized with highly expressed TF. In radioligand therapy, tumor growth was notably suppressed in the PEI + 0.45 mCi ^177^Lu-DOTA-tTF and PEI + 0.75 mCi ^177^Lu-DOTA-tTF groups compared to the PEI alone group (**Figure 6**). This observation underscored that TF as an ablation-triggered target pool at the ablation area boundary post-PEI facilitates delivery of ^177^Lu-DOTA-tTF to the interface between the ablation area and residual tumor, synergistically eliminating M2 macrophages and residual tumor cells.

## Conclusion

Al^18^F-NOTA-tTF showed potential as a PET tracer for monitoring the PEI ablation response in hepatocellular carcinoma, while ^177^Lu-DOTA-tTF served as an effective modality for inhibiting residual tumor growth and reducing the presence of immunosuppressive macrophages following PEI. Significantly, TF-targeting theranostics may provide a strategy to mitigate the challenges associated with incomplete cancer cell eradication and establish an immunosuppressive tumor microenvironment in HCC ablation. These findings suggest potential avenues for further clinical application, although additional studies are necessary to evaluate their effectiveness in a clinical setting.

## Supplementary Material

Supplementary materials and methods, figures.

## Figures and Tables

**Table 1:** Quality control of the Al^18^F-NOTA-tTF

Parameter	Result
Appearance	Colorless
pH	6.9-7.1
Radio-thin-layer chromatography	> 99%
Radio-HPLC	> 99%
Specific activity (MBq/nmol)	8.8-23.4
Ethanol	< 5%
Vitro stability in PBS	> 95% (3 h)
Vitro stability in FBS	> 95% (3 h)
Solubility	Hydrophilic

HPLC: high-performance liquid chromatography; PBS: phosphate-buffered saline; FBS: fetal bovine serum;

## Abbreviations

TF: tissue factor
tTF: targeting tissue factor
PEI: percutaneous ethanol injection
HCC: hepatocellular carcinoma
TACE: transarterial chemoembolization
RFA: radiofrequency ablation
IRE: irreversible electroporation
TIME: tumor immunosuppressive microenvironment
CT: computed tomography
PET: positron emission tomography
%ID/g: percentage of injected dose per gram
IHC: immunohistochemistry
HPLC: high-performance liquid chromatography
SD: standard deviation
