# Supplementary material for: Preclinical evaluation of radiolabeled tissue factor-targeted peptide for theranostics of hepatocellular carcinoma post percutaneous ethanol injection
Source: Theranostics. 2024 Oct 28;14(19):7370–82. doi: 10.7150/thno.102130 (PMC11626952; doi:10.7150/thno.102130)
Supplement: Supplementary file 1 — Supplementary materials and methods, figures. [file thnov14p7370s1.pdf]

Supplementary materials

Preclinical evaluation of radiolabeled tissue factor targeted peptide for theranostics of hepatocellular carcinoma post percutaneous ethanol injection

Buchuan Zhang[1,2], Pei Wang[1,2], Qiaorong Chen[1,2], Yilin Yang[1,2], Feng Xiong[1,2], Guangfa Bao[1], Jingfei Yang[1], Ziqiang Wang[1], Huimin Zhou[1], Shuang Song[1], Sijuan Zou[1], Dong-Hyun Kim[3], Bo Yu[1,2*], Xiaohua Zhu[1*]

1. Department of Nuclear Medicine, Tongji Hospital, Tongji Medical College, Huazhong University of Science and Technology. Wuhan, China.

2. Department of Nuclear Medicine, Tongji Hospital, Tongji Medical College and State Key Laboratory for Diagnosis and Treatment of Severe Zoonotic Infectious Diseases, Huazhong University of Science and Technology. Wuhan, China.

3. Department of Radiology, Feinberg School of Medicine, Northwestern University. Chicago, IL 60611, USA.

Corresponding authors:

Professor Bo Yu

yuboxf@hotmail.com

Professor Xiaohua Zhu

evazhu@vip.sina.com

Materials and methods

General

All chemicals, reagents, and solvents were commercially purchased without additional purification. Anhydrous aluminum chloride ($AlCl_3$) and sodium acetate were procured from Sigma-Aldrich (Shanghai) Trading Co. Ltd. Trifluoroacetic acid (TFA) was sourced from Shanghai Aladdin Biochemical Technology Co., Ltd. The precursor NOTA/DOTA-tTF was synthesized by Trigoats Co., Ltd. Reagents such as phosphate-buffered saline (PBS), cell culture medium, fetal bovine serum (FBS), penicillin-streptomycin solution (PS), and trypsin for cell culture and subsequent experiments were obtained from Gibco (Thermo Fisher Scientific, China).

Cell culture and tumor models

The Hepa1-6 cancer cell line was obtained from the American Type Culture Collection. Hepa1-6 cells were cultured in Dulbecco's modified Eagle's medium (DMEM) supplemented with 10% FBS and 1% PS at 37 °C in a 5% carbon dioxide atmosphere.

All animal experiments were conducted in compliance with the Guide for the Care and Use of Medical Laboratory Animals (Ministry of Health, China). Male C57BL/6 mice, aged six to eight weeks, were purchased from Beijing Vital River Laboratory Animal Technology Co., Ltd. Hepa1-6 cells (0.1 ml; 5×10^6 cells) were subcutaneously inoculated into the right shoulder. Tumors on the right flank were allowed to grow to approximately 5–10 mm in diameter before the animals were used for subsequent experiments.

Radiolabeling and Quality Control

Briefly, $^{18}F^-$ was loaded onto a QMA cartridge (Waters Corporation, USA) and subsequently eluted with saline (0.3-0.5 mL). For radiolabeling, No-carrier-added $^{18}F^-$ in saline (0.1 mL, 1.85–

3.7 GBq), sodium acetate buffer (0.1 mL, 0.1 M, pH 4.0), and $AlCl_3$ (20 μL, 40 nmol) in sodium acetate buffer (0.1 M, pH 4.0) were mixed and allowed to react at room temperature for 5 min. Subsequently, the NOTA-tTF precursor (10 μL, 50 nmol) was added to the reaction mixture and heated at 110 °C for 15 min. The reaction mixture was then diluted with 10 mL of ultrapure water and purified using a Sep-Pak Light C18 cartridge (Waters Corporation, USA), which had been pre-conditioned with 5 mL of EtOH and 10 mL of H_2O. The Sep-Pak C18 Light cartridge was washed with H_2O (5 mL) and eluted with 0.8 mL of 80% EtOH to obtain the final product. The product was passed through a sterile 0.2 μm filter membrane and diluted with saline for further studies. The radiochemical purity of $Al^{18}F$-NOTA-tTF was analyzed using radio high-performance liquid chromatography (HPLC) equipped with a γ-detector (Waters Corporation, USA). Mobile phases consisted of H_2O (A) and acetonitrile (B) mixed with 0.1% trifluoroacetic acid. Radio-HPLC was performed with a linear A–B gradient (75%–50% B over 20 min) at a flow rate of 1 mL/min, monitored at 220 nm ultraviolet light.

^{68}Ga-DOTA-tTF radiolabeling was conducted following established protocols. Briefly, 2 mL of 0.05 M aqueous HCl containing ^{68}Ga was transferred into a reaction vial containing 20 nmol of DOTA-tTF and 1 mL of 1 M sodium acetate. The mixture was heated at 95 °C for 15 min with magnetic stirring. The crude product was purified by passing through a C18 Sep-Pak cartridge. Radiolabeling efficiency was assessed using radio-thin-layer chromatography with citrate solvent (0.1 M, pH 4.0) as the mobile phase.

The procedure for labeling DOTA-tTF with ^{177}Lu followed previously established protocols. After adding 300 MBq of [177Lu] $LuCl_3$ (Chengdu Xinke Pharmaceutical Co. Ltd, China) and 20 nmol of DOTA-tTF to the vial, the reaction pH was adjusted to 5 and the temperature was

increased to 95 °C, and maintained for 15 min. The crude product was purified using a C18 Sep-Pak cartridge and sterilized for subsequent applications. Radiolabeling efficiency was assessed using radio-thin-layer chromatography with citrate solvent (0.1 M, pH 4.0) as the mobile phase.

Small animal biodistribution and pharmacokinetics study of $Al^{18}F$-NOTA-tTF

A small animal biodistribution experiment was conducted with female Kunming mice (5 weeks). The mice were divided into six groups with 3-4 mice in each group. $Al^{18}F$-NOTA-tTF (1.2 MBq) was injected into KM mice via the tail vein and a 1% injected dose of $Al^{18}F$-NOTA-tTF was prepared for reference. At 5 min,15 min, 30 min, 60 min, 90 min and 120 min post-injection, the KM mice were euthanized and organs of interest were collected, wet-weighed. The uptake in various organs and tissues was determined and expressed in terms of the percentage injected dose per gram (%ID/g, mean ± SD) after quantifying the radioactivity of the samples using an automated γ-counter.

For the pharmacokinetic study, each Kunming mouse was intravenously injected with a 3.7 MBq of $Al^{18}F$-NOTA-tTF. Small amounts of blood were obtained through the orbital vein of the mice at 1, 3, 5, 10, 15, 30, 60, 90, 120, 180 and 240 min after injection. The blood samples were weighed and measured for radioactivity by the γ-counter. The results are expressed as the percentage injected dose per gram (%ID/g).

$Al^{18}F$-NOTA-tTF PET/CT imaging in mice bearing Hepa1-6 xenografts

$Al^{18}F$-NOTA-tTF PET/CT imaging of Hepa1-6 tumor-bearing mice were intravenously injected with 100 μL of $Al^{18}F$-NOTA-tTF (2.96 MBq). In the blocking experiments, unlabeled excess precursors (0.2 mg) were co-injected with $Al^{18}F$-NOTA-tTF into Hepa1-6 mice (n = 3). One hour after $Al^{18}F$-NOTA-tTF injection, the mice were anesthetized and placed in the prone

position on the scanning bed. The PET/CT imaging was acquired with a PET/CT scanner (uBio-EXPLORER, United Imaging, China). PET images were collected for 10 min and reconstructed to get more high-resolution images using an ordered subsets expectation maximization (OSEM) algorithm (4 OSEM iterations, resolution: 1.4 mmHD) with scatter, attenuation, and decay corrections applied. Regions of interest (ROIs) were drawn on the CT images and further mapped on PET. The SUV_{max} of the tumor and muscle were calculated and referred to as the tumor-to-muscle ratio.

PEI treatment and efficacy monitoring

In this study, we established a model of incomplete ablation of hepatocellular carcinoma using percutaneous ethanol injection (PEI). Once the tumor volume reached approximately 100 mm^3, all mice were randomly assigned to treatment, sham treatment, and negative control groups. During the PEI procedure, an insulin needle was inserted along the long diameter of the tumor, with needle depth set at 1/2 to 2/3 of the tumor diameter. Ethanol was injected at a rate of 25 μL every half minute, and the needle was slowly withdrawn after 5 minutes of injection. Ethanol leakage from the tumor was assessed using CT scans immediately following PEI treatment, defined as day 0. Subsequently, ^{18}F-FDG PET/CT imaging was performed on day 1 post-PEI to evaluate therapeutic efficacy. Tumor volume and mouse body weight were monitored every 2 to 3 days. Tumor volume was calculated using the formula volume = (length × width^2)/2.

Radioligand therapy study

Mice were randomly assigned to five groups: PEI (group 1, n = 10), PEI + 0.45 mCi ^{177}Lu-DOTA-tTF (group 2, n = 10), PEI + 0.75 mCi ^{177}Lu-DOTA-tTF (group 3, n = 8), 0.45 mCi ^{177}Lu-DOTA-tTF (group 4, n = 10), and saline (group 5, n = 10). During radiotherapy, one mouse died in

groups 2 and 4 due to unknown reasons, and four mice died in group 3 due to low temperature in the imaging scanning room and anesthesia factors.

Evaluation of safety and tolerability of PEI

On day 2 and day 10 after PEI, whole blood and mouse serum were collected, and blood routine indexes, alanine aminotransferase (ALT), aspartate aminotransferase (AST) and blood urea nitrogen (BUN) levels were determined. Hematoxylin-eosin-stained sections of the heart, liver, spleen, lungs, and kidneys were used to assess tissue damage.

Evaluation of safety and tolerability of radiation therapy

To assess the safety and tolerability of radiation therapy, blood counts and liver and kidney function tests were performed. The heart, liver, spleen, lungs and kidneys were stained with hematoxylin and eosin (H&E) to evaluate for tissue damage.

Figures

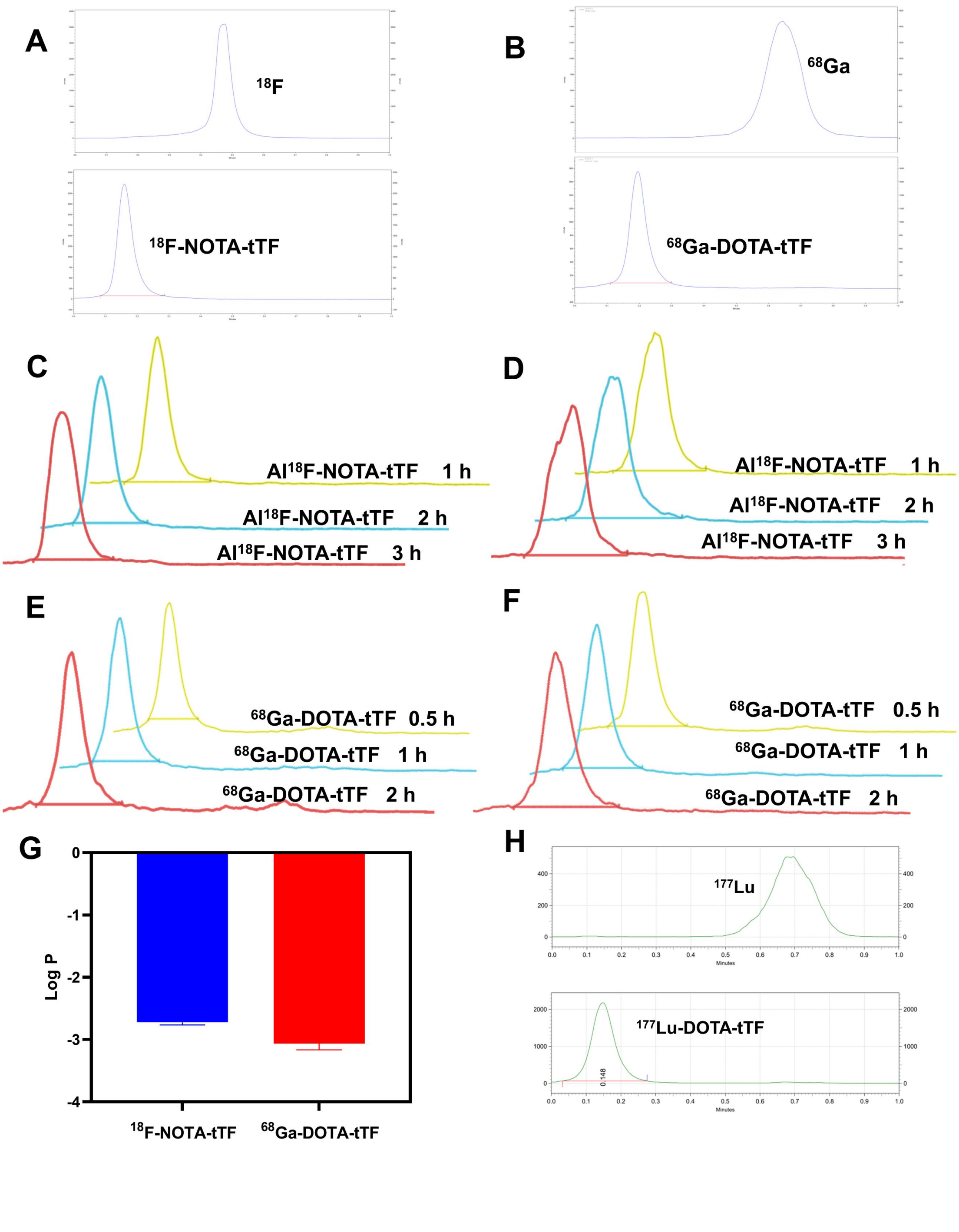

Figure S1. Quality control results of the probes.

The radiochemical purity of Al^{18}F-NOTA-tTF**(A)**, ^{68}Ga-DOTA-tTF**(B)**, ^{177}Lu-DOTA-tTF**(H)** via

instant thin layer chromatography. The stability of $Al^{18}F$-NOTA-tTF in PBS **(C)** and FBS **(D)** via instant thin layer chromatography. The stability of ^{68}Ga-DOTA-tTF in PBS **(E)** and FBS **(F)** via instant thin layer chromatography. **(G)** The Log P values of $Al^{18}F$-NOTA-tTF and ^{68}Ga-DOTA-tTF. FBS: fetal bovine serum; PBS: phosphate-buffered saline.

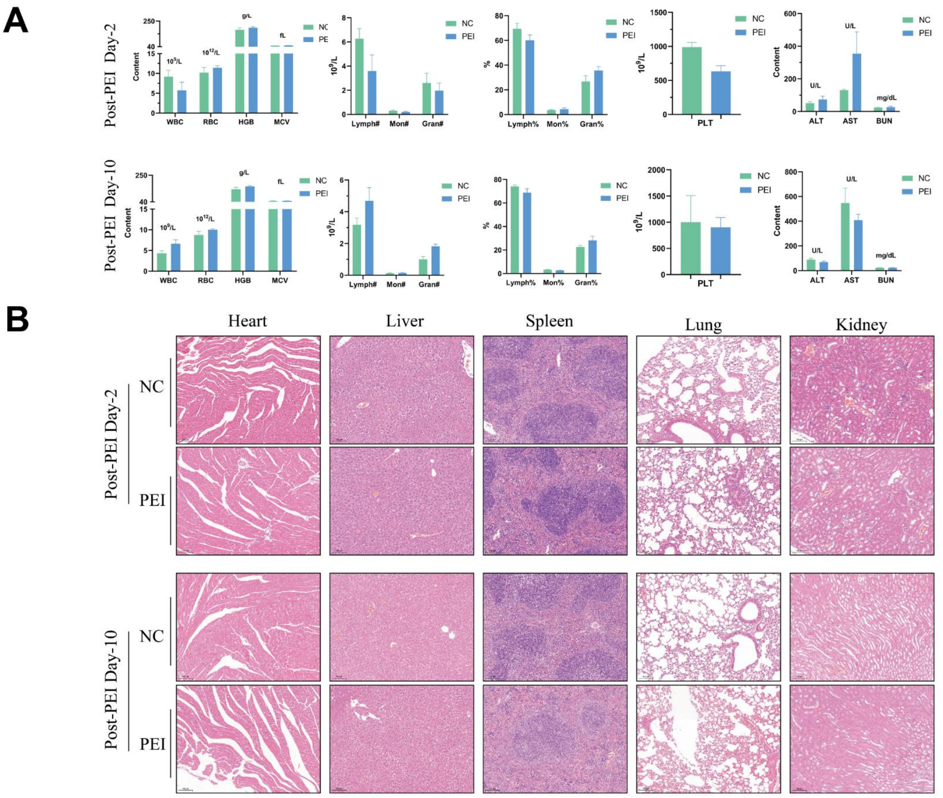

Figure S2. Evaluation of safety and tolerability of PEI.

(A). Blood count tests and liver and kidney function tests were conducted on mice in the PEI group and the negative control (NC) group on day 2 and day 10 post-PEI. The parameters measured included RBC (Red Blood Cell), WBC (White Blood Cell), PLT (Platelet), HGB (Hemoglobin), MCV (Mean Corpuscular Volume), Lymph# (Lymphocyte count), Mon# (Monocyte count), Gran# (Granulocyte count), Lymph% (Lymphocyte percentage), Mon% (Monocyte percentage), Gran% (Granulocyte percentage), ALT (Alanine Transaminase), AST (Aspartate Aminotransferase), and BUN (Blood Urea Nitrogen). **(B).** H&E staining of healthy organs from mice in the PEI group and the NC group on day 2 and day 10 post-PEI.

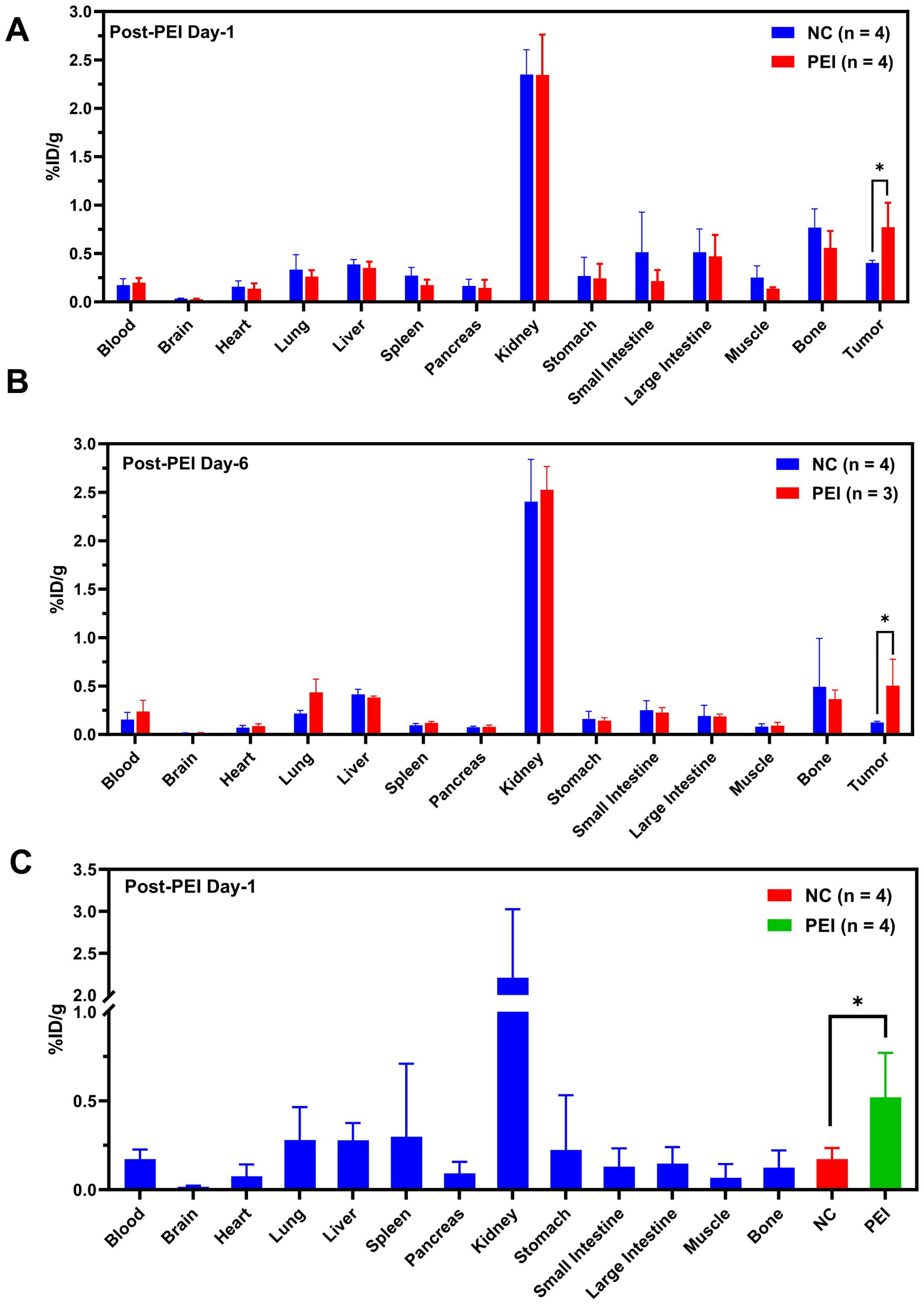

Figure S3. Biodistribution study of radioactive probes after PEI treatment.

Biodistribution of $Al^{18}F$-NOTA-tTF at 1 h post injection in Hepa1-6 subcutaneous xenograft models on day 1 **(A)** and day 6 **(B)** after PEI treatment. Biodistribution of ^{68}Ga-DOTA-tTF at 1 h post injection in Hepa1-6 subcutaneous xenograft models on day 1 **(C)** after PEI treatment. The data are expressed as the mean ± SD.

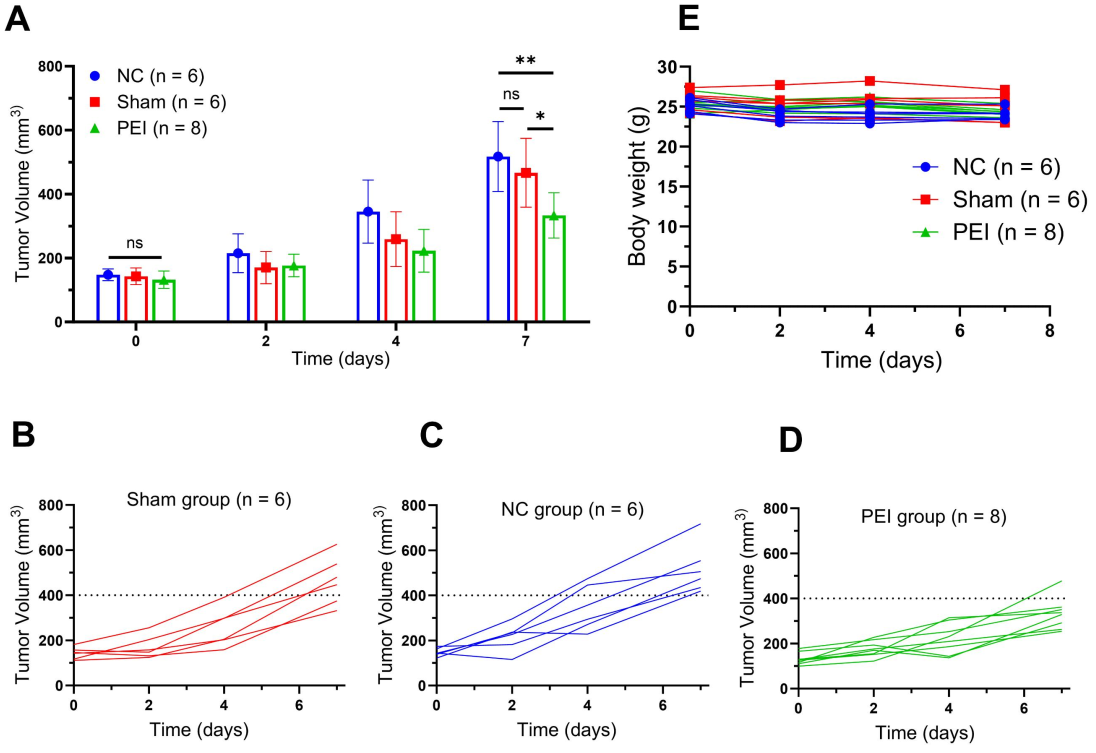

Figure S4. PEI treatment and efficacy monitoring in Hepa1-6 mice.

(A) Tumor growth curve of each group. **(B-D)** Tumor growth curves of the Sham group (n = 6), NC group (n = 6) and PEI group (n = 8), respectively. **(E)** Body weight change of Hepa1-6 tumor-bearing mice.

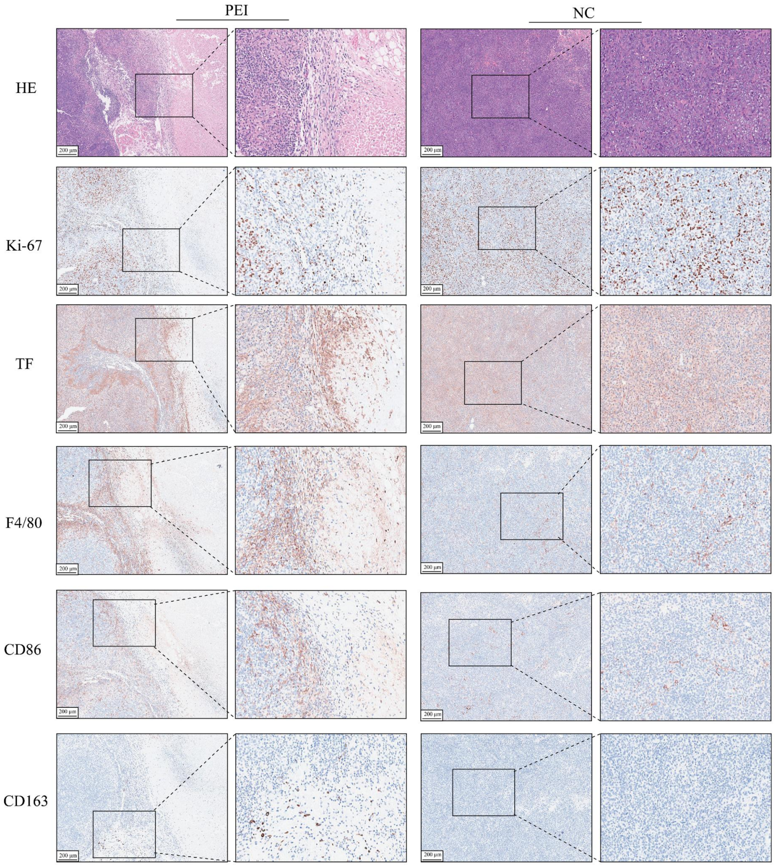

Figure S5. Histopathological results of tumor from Hepa1-6 tumor-bearing mice on day 6 post-PEI.

Tumor H&E and immunohistochemical staining of Ki-67 and tissue factor on day-6 after PEI treatment. The data are expressed as the mean ± SD. Scale bar = 200 μm.

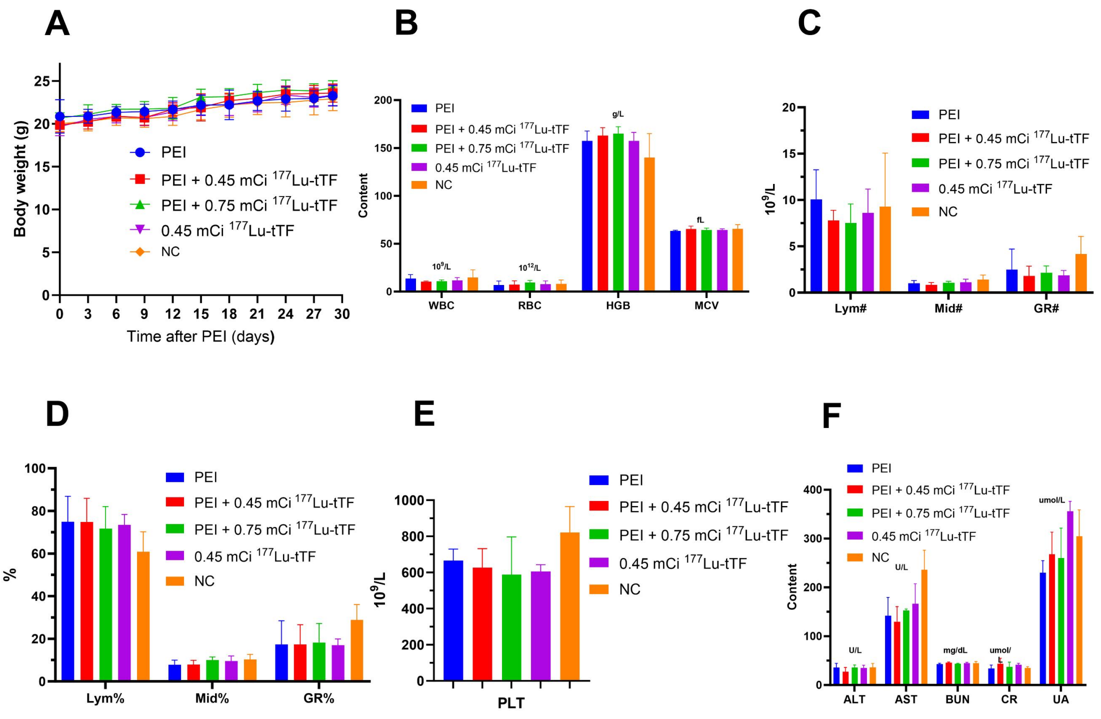

Figure S6. Safety and tolerability evaluation of radiation therapy.

(A) Body weight change of tumor-bearing mice during the therapy study. **(B-F)** Blood cell count and liver and kidney function test for mice of each group on day-29 after ^{177}Lu-DOTA-tTF administration. RBC: Red blood cell; WBC: White blood cell; PLT: Platelet; HGB: Hemoglobin; MCV: Mean corpuscular volume; Lymph#: Lymphocyte; Mid#: Monocyte; GR#: Granulocyte; Lymph%: Lymphocyte percentage; Mid%: Monocyte percentage; GR%: Granulocyte percentage; ALT: Alanine transaminase; AST: Aspartate aminotransferase; BUN: Blood urea nitrogen; CR: Creatinine; UA: Uric acid.

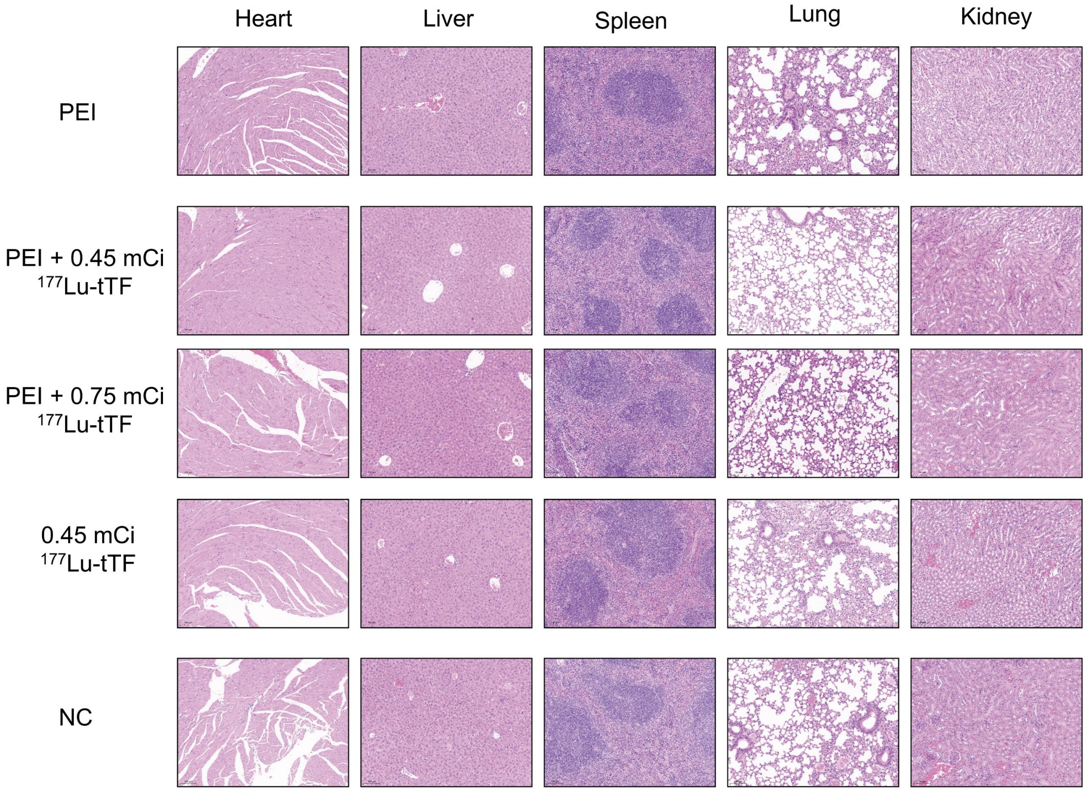

Figure S7. Safety and tolerability evaluation of radiation therapy.

During radiotherapy, H&E staining of healthy organs from mice of each group on day-29 after PEI treatment displayed no obvious abnormalities. Scale bar = 100 μm.
